# LTE RSRP, RSRQ, RSSNR and local topography profile data for RF propagation planning and network optimization in an urban propagation environment

**DOI:** 10.1016/j.dib.2018.08.137

**Published:** 2018-08-31

**Authors:** Oluyomi Simpson, Yichuang Sun

**Affiliations:** School of Engineering and Technology, University of Hertfordshire, Hatfield AL10 AB, United Kingdom

**Keywords:** LTE, Reference Signal Received Power (RSRP), Reference Signal Received Quality (RSRQ), Reference Signal Signal to Noise Ratio (RSSNR), RF propagation planning, RF network optimization

## Abstract

In the design of 5 G cellular communication to guarantee quality signal reception at every point within a coverage area, fundamental knowledge of the channel propagation characteristics is vital. A correct knowledge of electromagnetic wave propagation is required for efficient radio network planning and optimization. Propagation data are used extensively in network planning, particularly for conducting feasibility studies. Hence, measurement of accurate propagation models that predict how the channel varies as people move about is crucial. However, these measured data are often not widely available for channel characterization and propagation model development. In this data article, the Reference Signal Received Power (RSRP), Reference Signal Received Quality (RSRQ) and Reference Signal Signal to Noise Ratio (RSSNR) at various points in space which is covered by a Long-Term Evolution (LTE) marco base station operating at 2100 M*Hz* located in Hatfield, Hertfordshire, United Kingdom were measured. Further, local topography profile data of the study area were extracted from a digital elevation model (DEM) to account for the features of the propagation environment. Correlation matrix and descriptive statistics of the measured LTE data along different routes are analyzed. The RSRP, RSRQ and RSSNR variation with transmitter (Tx) – receiver (Rx) separation distance along the routes are presented. The probability distribution and the DEM of LTE data measurement are likewise presented. The data provided in this article will facilitate research advancement in wireless channel characterization that accounts for local topography features in an urban propagation environment. Moreover, the data sets provided in this article can be extended using simulation-based analysis to extract spatial and temporal channel model parameters in urban cellular environments in the development of 5 G channel propagation models.

**Specifications table**

**Table:** 

Subject area	*Engineering*
More specific subject area	*Wireless and Mobile Communication Engineering*
Type of data	*Tables, graphs, figures, spreadsheet file (.xlsx), map file (.kml)*
How data was acquired	– *LTE receiver field measurement data was collected over a LTE marco base station operating at 2100 MHz using a test reconfigurable Base Transceiver System (BTS).* – *The BTS is based on Software Defined Radio (SDR) using a National Instrument (NI) Universal Software Radio Peripheral (USRP) B200 board and OpenBTS.* – *OpenBTS was operated using open source software GNU Radio running on a Linux OS.* – *Global Position System׳s (GPS) Latitude and longitude data were collected using the USRP.* – *Local topography profile data were obtained from Shuttle Radar Topography Mission (SRTM1) dataset.*
Data format	*Raw and analyzed*
Experimental factors	– *The RF measurements were carried out under good climatic conditions.* – *An average speed of 20 mile per hour by the vehicle was maintained throughout the propagation measurement along the drive route.*
Experimental features	– *Correlation matrix and descriptive statistics of measured LTE data and local topography profile data are presented.* – *Measured LTE data variation with respect to slot and Tx – Rx separation.* – *Probability distribution of measured LTE data measurement are presented.* – *The digital elevation model (DEM) of measured LTE data are presented.*
Data source location	*The LTE measurement and local topography profile data presented in this article were collected in Hatfield, Hertfordshire, United Kingdom (Latitude 51° 44׳ 56.72" N and longitude 000° 14׳ 33.65" W).*
Data accessibility	*Datasets on various measurements such as RSRP, RSRQ, RSSNR, Tx- Rx Distance and Altitude are provided with this article.*

**Value of the data**

• The data provided in this article will facilitate research advancement in wireless channel characterization that accounts for local topography features in an urban university campus propagation environment.
• The data provided in this article will provide useful insights into the performance of cellular networks under different fading conditions, during the network planning and for designing future 5 G network infrastructure to ensure an adequate quality-of-service for all users in an urban university campus propagation environment.
• The data will facilitate research development of analytical standard models such as the 3rd Generation Partnership Project (3GPP) WINNER II MIMO channel model for long term evolution (LTE)-Advanced and other proposed models for future 5 G systems for sub−6 GHz and mmWave frequencies.

• The data sets provided can be extended using simulation-based analysis to extract spatial and temporal channel model parameters in urban cellular environments in the development of 5 G channel propagation models.

## 1 Data

To meet the ever-increasing demand for data on the move, all major telecommunications companies, as well as global standardization entities, are actively driving the research and development of 5 G cellular communications [1], [2]. During the deployment of 5 G cellular communication to increase cellular network capacity, cellular base station will need to be upgraded [1]. Theses base station features will include a new generation of high-capacity base band units, multi-band remote radio units, Large-bandwidth and high-power C-band Massive MIMO active antenna unit, and high-power cabinets [3].

In the design of 5 G cellular communication to guarantee quality signal reception at every point within the coverage area, fundamental knowledge of the channel propagation characteristics is vital. A correct knowledge of electromagnetic wave propagation is also required for efficient radio network planning and optimization [4]. Propagation data are used extensively in network planning, particularly for conducting feasibility studies. They are also very useful for performing interference studies as the deployment proceeds [5].

**Fig. 1:**
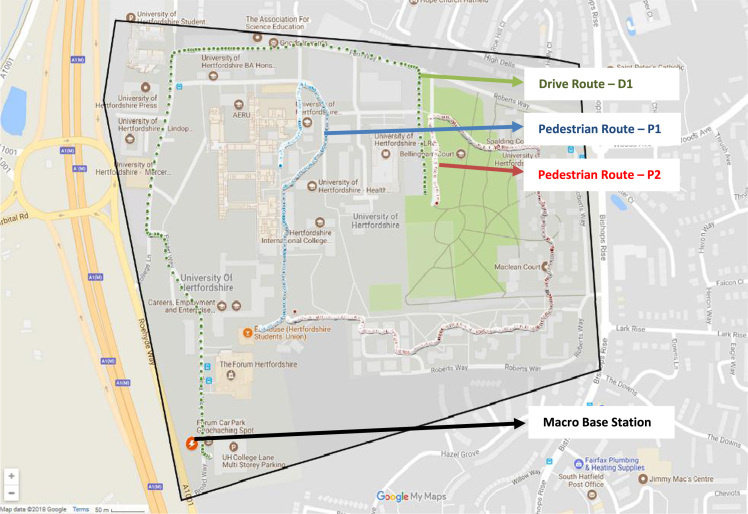
Data collection region and complete measurement routes followed [7].

**Fig. 2:**
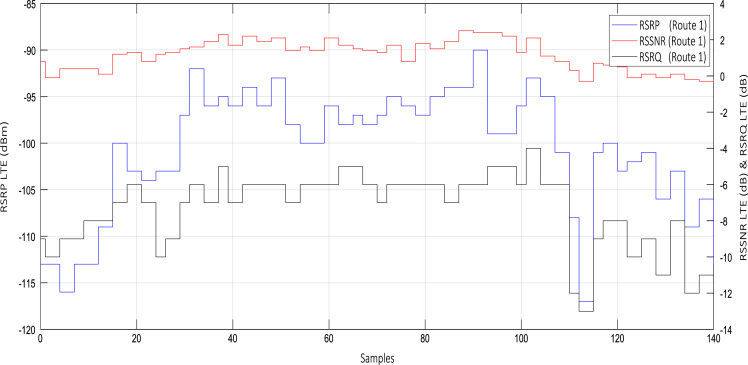
RSRP (dBm), RSRQ (dB) and RSSNR (dB) variation along drive route – D1 (route 1).

**Fig. 3:**
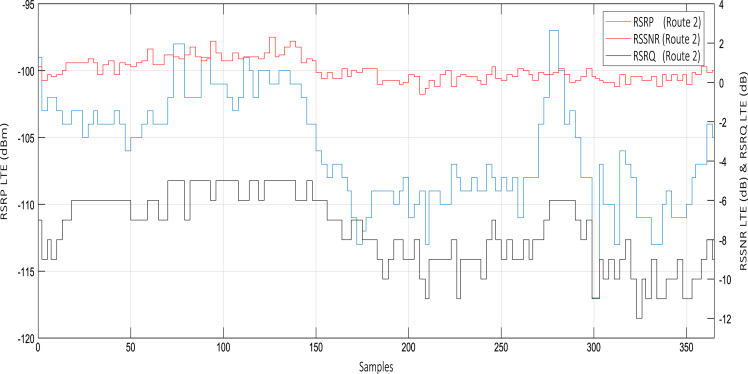
RSRP (dBm), RSRQ (dB) and RSSNR (dB) variation along pedestrian route – P1 (route 2).

**Fig. 4:**
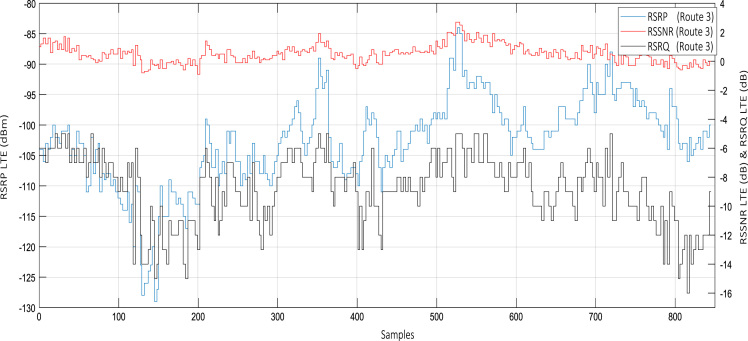
RSRP (dBm), RSRQ (dB) and RSSNR (dB) variation along pedestrian route – P2 (route 3).

**Fig. 5:**
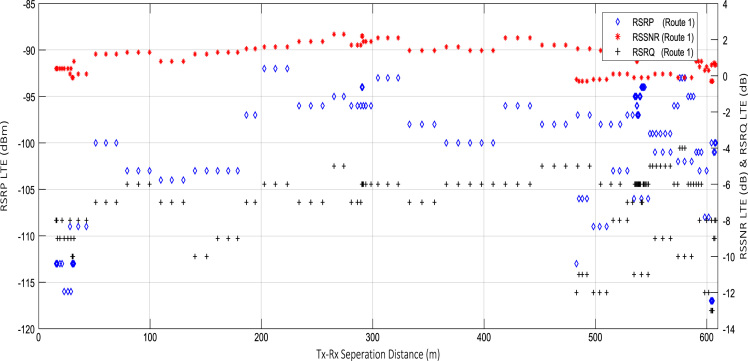
RSRP (dBm), RSRQ (dB) and RSSNR (dB) vs Tx–Rx Separation Distance (m) along drive route – D1 (route 1).

**Fig. 6:**
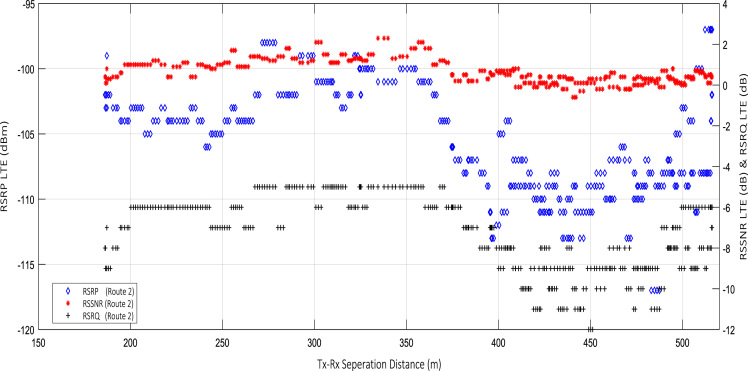
RSRP (dBm), RSRQ (dB) and RSSNR (dB) vs Tx–Rx Separation Distance (m) along pedestrian route – P1 (route 2).

**Fig. 7:**
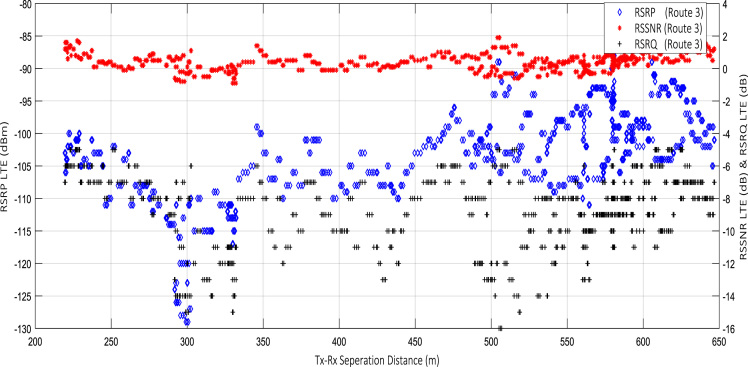
RSRP (dBm), RSRQ (dB) and RSSNR (dB) vs Tx–Rx Separation Distance (m) along pedestrian route – P2 (route 3).

**Fig. 8:**
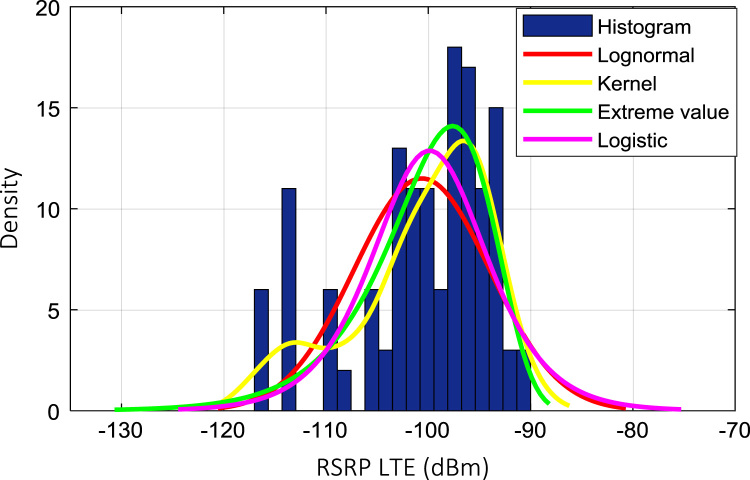
RSRP (dBm) probability distribution along drive route – D1 (route 1).

**Fig. 9:**
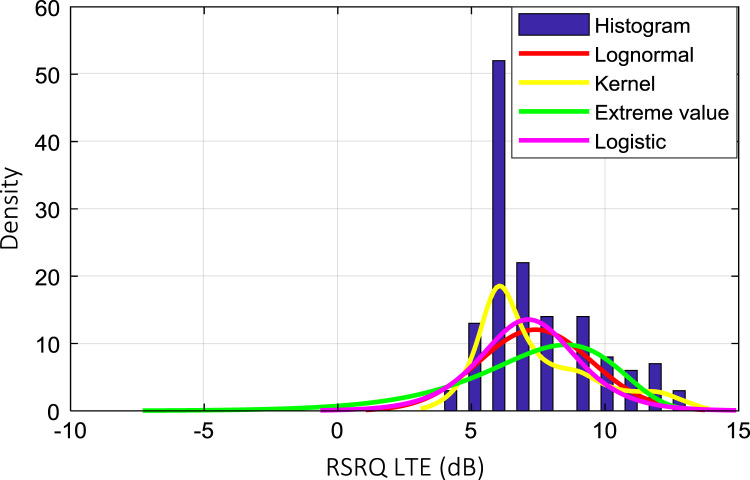
RSRQ (dB) probability distribution along pedestrian route – P1 (route 1).

**Fig. 10:**
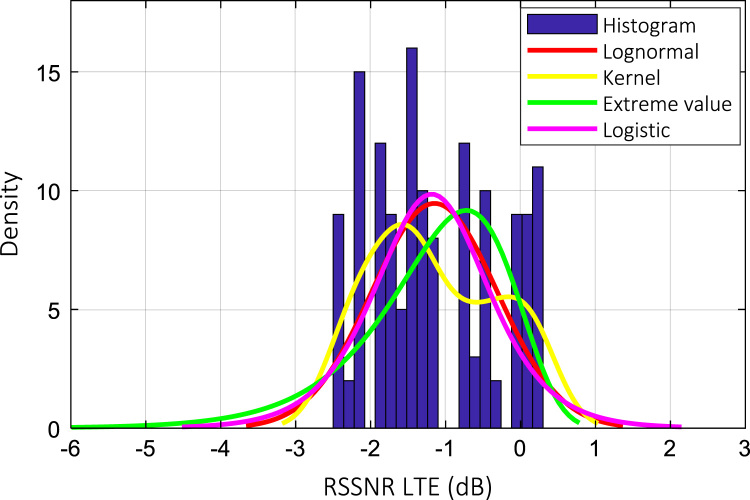
RSSNR (dB) probability distribution along pedestrian route – P2 (route 1).

**Fig. 11:**
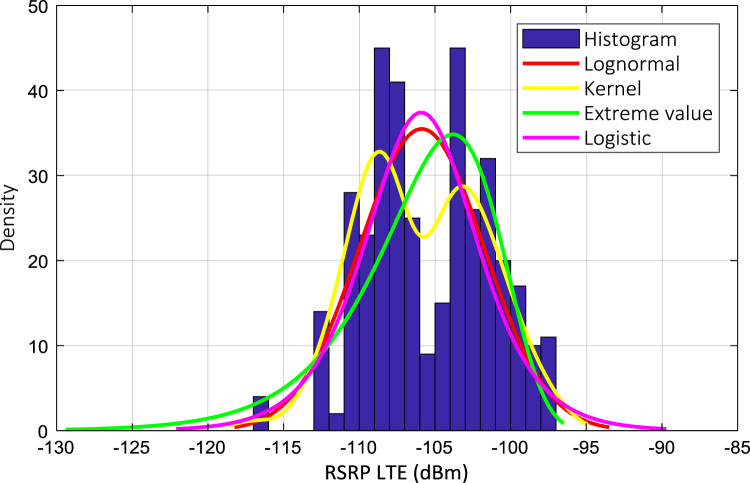
RSRP (dBm) probability distribution along drive route – D1 (route 2).

**Fig. 12:**
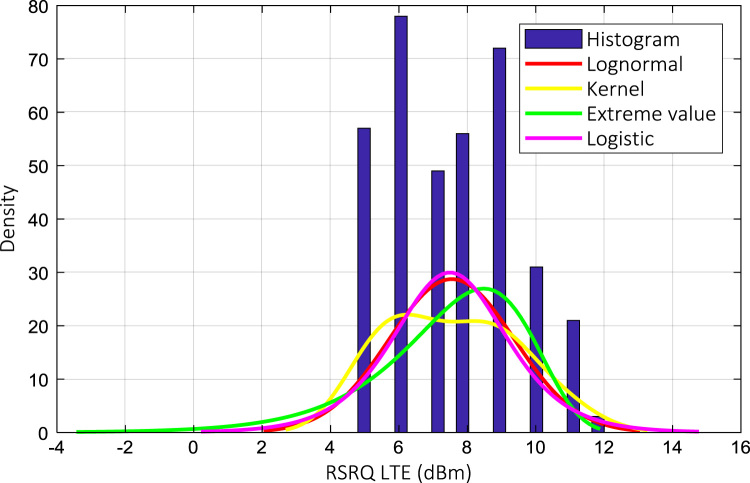
RSRQ (dB) probability distribution along pedestrian route – P1 (route 2).

**Fig. 13:**
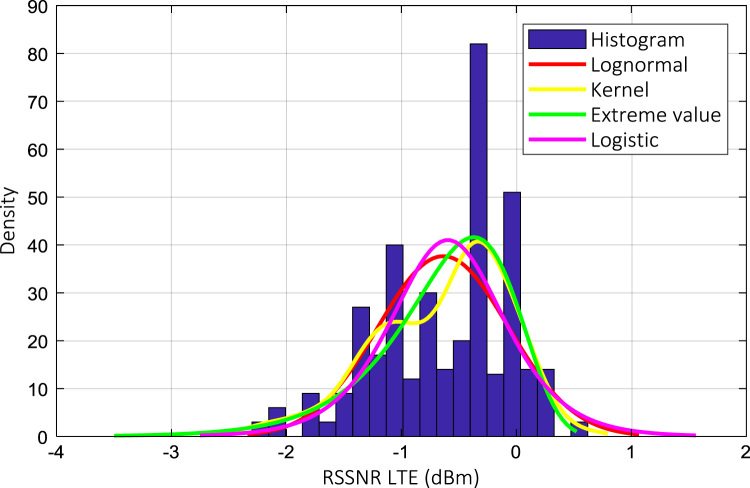
RSSNR (dB) probability distribution along pedestrian route – P2 (route 2).

**Fig. 14:**
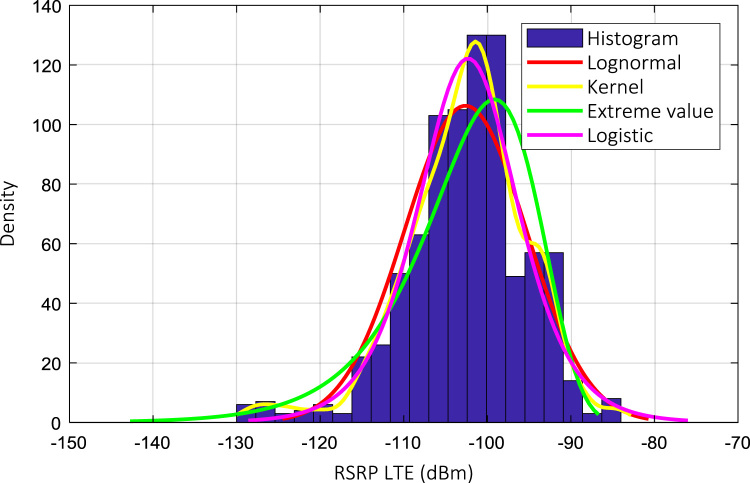
RSRQ (dBm) probability distribution along drive route – D1 (route 3).

**Fig. 15:**
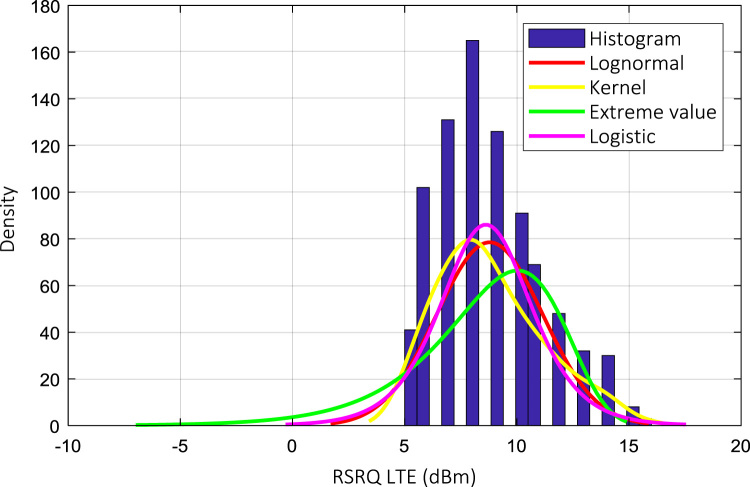
RSRQ (dB) probability distribution along pedestrian route – P1 (route 3).

**Fig. 16:**
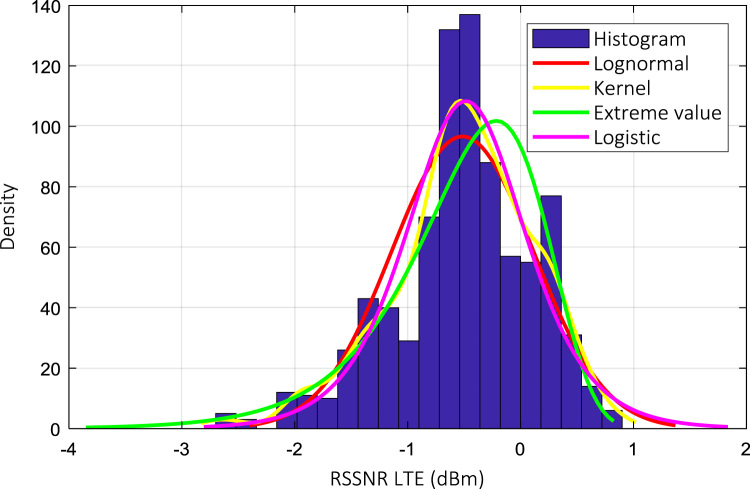
RSSNR (dB) probability distribution along pedestrian route – P2 (route 3).

**Fig. 17:**
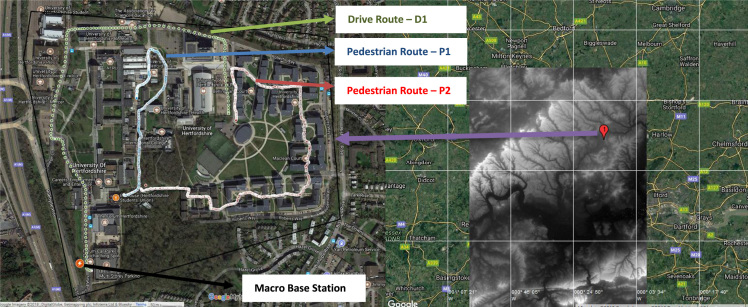
Digital elevation model topography map [7], [8].

**Fig. 18:**
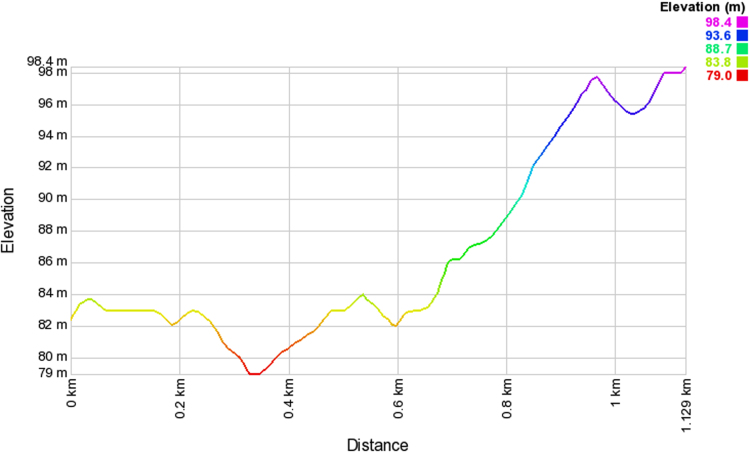
Digital elevation model along drive route – D1 (route 1).

**Fig. 19:**
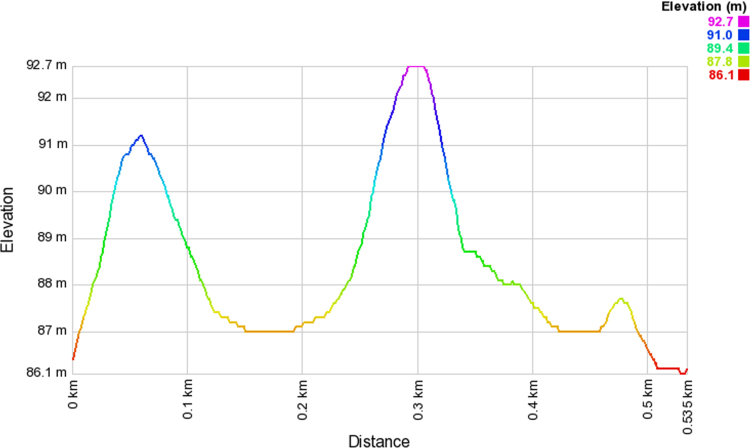
Digital elevation model along pedestrian route – P2 (route 2).

**Fig. 20:**
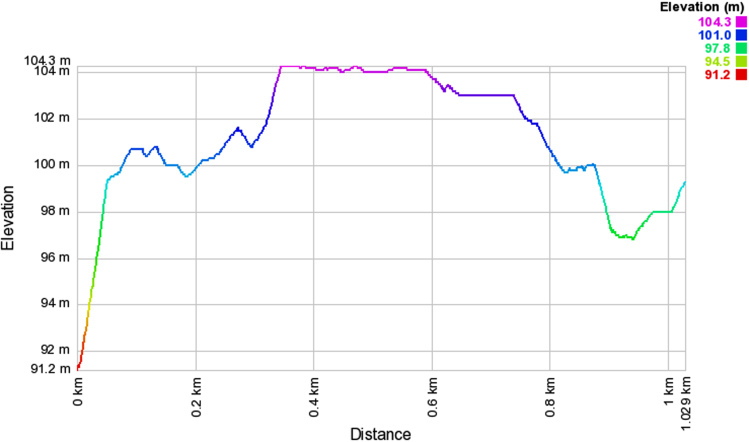
Digital elevation model along pedestrian route – P2 (route 3).

Wireless communications engineers rely on measurement data and local terrain profile information to determine optimal locations of base stations; attain best possible data rates; predict radio coverage; determine the required Tx power; aid appropriate selection of antenna height and pattern; conduct radio network optimization; perform interference feasibility studies; and ensure an acceptable level of quality of service without the need of expensive and time consuming measurements [6]. In this data article, the Reference Signal Received Power (RSRP), Reference Signal Received Quality (RSRQ) and Reference Signal Signal to Noise Ratio (RSSNR) from a LTE Marco base station operating at frequency 2100 M*Hz* located in Hatfield, Hertfordshire, United Kingdom (Latitude 51° 44׳ 56.72" N and longitude 000° 14׳ 33.65" W) were measured along a drive route D1 and 2 pedestrian routes P1 and P2 as shown in Fig. 1. Correlation matrix and descriptive statistics of measured LTE data along drive D1 (route 1), pedestrian P1 (route 2) and pedestrian P2 (route 3) are present in Table 1, Table 2, Table 3, respectively. Fig. 2, Fig. 3, Fig. 4 represent the RSRP, RSRQ and RSSNR variation in N – Sample slots along route 1, route 2 and route 3, respectively. The RSRP, RSRQ and RSSNR variation with transmitter (Tx) – receiver (Rx) separation distance along route 1 – 3, respectively are presented in Fig. 5, Fig. 6, Fig. 7. Fig. 8, Fig. 9, Fig. 10, Fig. 11, Fig. 12, Fig. 13, Fig. 14, Fig. 15, Fig. 16 present the probability distribution of RSRP, RSRQ and RSSNR LTE data measurement along route 1 – 3, respectively. In Fig. 17, Fig. 18, Fig. 19, Fig. 20, the digital elevation model (DEM) of RSRP, RSRQ and RSSNR measured data along route 1–3, respectively, are shown in. The DEM terrain is presented in Fig. 17.

**Table 1:** Correlation matrix and descriptive statistics of measured LTE data along drive route – D1 (*n* = 142).

*Route 1 - D1*	**RSRP (dBm)**	**RSRQ (dB)**	**RSSNR (dB)**	**Tx- Rx Distance (m)**	**Altitude (m)**
**RSRP (dBm)**	1.00000				
**RSRQ (dB)**	0.76603	1.00000			
**RSSNR (dB)**	0.79865	0.82967	1.00000		
**Tx- Rx Distance (m)**	0.35399	0.04587	0.05834	1.00000	
**Altitude (m)**	−0.34087	−0.56812	−0.62685	0.59098	1.00000
**Mean**	−100.62676	−7.39437	1.14789	394.24706	86.66127
**Standard Error**	0.55796	0.17737	0.07032	16.68969	0.53384
**Median**	−99.00000	−7.00000	1.30000	493.65500	83.20000
**Mode**	−96.00000	−6.00000	0.80000	482.96600	83.00000
**Standard Deviation**	6.64885	2.11364	0.83791	198.88057	6.36145
**Sample Variance**	44.20722	4.46749	0.70209	39,553.47970	40.46806
**Kurtosis**	−0.05214	0.12849	−1.14921	−0.96960	−0.99506
**Skewness**	−0.86603	−0.95839	−0.24912	−0.71951	0.77333
**Range**	27.00000	9.00000	2.80000	592.66300	19.40000
**Minimum**	−117.00000	−13.00000	−0.30000	15.49900	79.00000
**Maximum**	−90.00000	−4.00000	2.50000	608.16200	98.40000
**Sum**	−14,289.00000	−1050.00000	163.00000	55,983.08300	12,305.90000
**Count**	142.00000	142.00000	142.00000	142.00000	142.00000
**Confidence Level (95.0%)**	1.10305	0.35065	0.13901	32.99437	1.05537

*Note*. All correlation were significant at *p* < .01. Tx = Transmitter: Rx = Receiver.

**Table 2:** Correlation matrix and descriptive statistics of measured LTE data along pedestrian route – P1 (n=367).

*Route 2 - P1*	**RSRP (dBm)**	**RSRQ (dB)**	**RSSNR (dB)**	**Tx- Rx Distance (m)**	**Altitude (m)**
**RSRP (dBm)**	1.00000				
**RSRQ (dB)**	0.79846	1.00000			
**RSSNR (dB)**	0.67556	0.75220	1.00000		
**Tx- Rx Distance (m)**	−0.54251	−0.53318	−0.55844	1.00000	
**Altitude (m)**	−0.24042	−0.08427	−0.20733	−0.05408	1.00000
**Mean**	−105.86921	−7.54496	0.63651	380.08749	88.36458
**Standard Error**	0.21536	0.09573	0.02963	5.21768	0.09162
**Median**	−106.00000	−7.00000	0.50000	404.67500	87.70000
**Mode**	−104.00000	−6.00000	0.40000	187.15400	87.00000
**Standard Deviation**	4.12566	1.83396	0.56764	99.95636	1.75524
**Sample Variance**	17.02110	3.36341	0.32222	9991.27446	3.08087
**Kurtosis**	−0.63911	−0.94638	−0.07042	−1.06125	−0.02334
**Skewness**	−0.00388	−0.25923	0.59754	−0.43682	0.99543
**Range**	20.00000	7.00000	2.90000	329.92800	6.60000
**Minimum**	−117.00000	−12.00000	−0.60000	186.16700	86.10000
**Maximum**	−97.00000	−5.00000	2.30000	516.09500	92.70000
**Sum**	−38,854.00000	−2769.00000	233.60000	139,492.10700	32,429.80000
**Count**	367.00000	367.00000	367.00000	367.00000	367.00000
**Confidence Level (95.0%)**	0.42349	0.18825	0.05827	10.26039	0.18017

*Note*. All correlation were significant at *p* < .01. Tx = Transmitter: Rx = Receiver.

**Table 3:** Correlation matrix and descriptive statistics of measured LTE data along pedestrian route – P2 (n=846).

*Route 2 - P1*	**RSRP (dBm)**	**RSRQ (dB)**	**RSSNR (dB)**	**Tx- Rx Distance (m)**	**Altitude (m)**
**RSRP (dBm)**	1.00000				
**RSRQ (dB)**	0.53390	1.00000			
**RSSNR (dB)**	0.56762	0.74420	1.00000		
**Tx- Rx Distance (m)**	0.62252	0.04869	0.24856	1.00000	
**Altitude (m)**	0.05989	0.07969	0.17529	0.53149	1.00000
**Mean**	−102.60875	−8.79078	0.51418	477.06914	100.87045
**Standard Error**	0.25099	0.08127	0.02161	4.54318	0.10400
**Median**	−102.00000	−8.00000	0.50000	527.82600	100.80000
**Mode**	−101.00000	−8.00000	0.40000	561.14400	103.00000
**Standard Deviation**	7.30044	2.36392	0.62843	132.14319	3.02497
**Sample Variance**	53.29644	5.58813	0.39492	17,461.82247	9.15042
**Kurtosis**	1.33550	−0.14329	0.51639	−1.07435	0.93493
**Skewness**	−0.64380	−0.61733	0.55765	−0.61897	−1.02304
**Range**	45.00000	11.00000	3.60000	427.59900	13.10000
**Minimum**	−129.00000	−16.00000	−0.90000	219.36400	91.20000
**Maximum**	−84.00000	−5.00000	2.70000	646.96300	104.30000
**Sum**	−86,807.00000	−7437.00000	435.00000	403,600.48952	85,336.40000
**Count**	846.00000	846.00000	846.00000	846.00000	846.00000
**Confidence Level (95.0%)**	0.49265	0.15952	0.04241	8.91723	0.20413

*Note*. All correlation were significant at *p* < .01. Tx = Transmitter: Rx = Receiver.

## 2 Experimental design, materials, and methods

LTE radio resource management measurement was conducted within an urban university campus - Hatfield, Hertfordshire, United Kingdom. The propagation environment is a typical urban area comprising of distributed buildings of various heights, vegetation and open lands. Three routes covered by a macro base station were mapped out as shown in Fig. 1 and Fig. 17. The macro base station has an antenna height of 15 m, Tx power of 28.7 dBW and operating frequency of 2100 MHz.

The LTE receiver field measurement data was collected using a test reconfigurable Base Transceiver System (BTS). The BTS is based on Software Defined Radio (SDR) using a National Instrument (NI) Universal Software Radio Peripheral (USRP) B200 board and OpenBTS. A retractable 9 dBi omni-directional whip antenna was coupled to the USRP. The network testing software OpenBTS was realized using open source software GNU Radio running on a Linux OS. The Linus OS was running on a 7th generation Intel® Core™ i7–7500U CPU processor with 16 GB RAM. The Global Position System׳s (GPS) Latitude and longitude data were collected using the USRP with a magnetic mount GPS antenna attached to the USRP for enhanced functionality. The local topography profile data were obtained from NASA׳s SRTM1 database [8] digital terrain map. For route 1 measurements the setup was placed in a vehicle driven at an average speed of 20 mile per hour. The speed of the vehicle was maintained throughout the propagation measurement along D1 (route 1). For the pedestrian test the setup was loaded into a Portable walking safety laptop desk harness for both P1 (route 2) and P2 (route 3). All measurements were carried out under good climatic conditions.

### 2.1 Correlation matrix and descriptive statistics of measured LTE data

 See Table 1, Table 2, Table 3.

### 2.2 Measured LTE data variation

 See Fig. 2, Fig. 3, Fig. 4, Fig. 5, Fig. 6, Fig. 7.

### 2.3 Probability distribution of measured LTE data measurement

 See Fig. 8, Fig. 9, Fig. 10, Fig. 11, Fig. 12, Fig. 13, Fig. 14, Fig. 15, Fig. 16.

### 2.4 Digital elevation model (DEM) of measured LTE data

 See Fig. 17, Fig. 18, Fig. 19, Fig. 20.

## Appendix A Supplementary materials

The following KMZ files contain the Google maps of the most important areas described in this article.

Map KMZ file containing the Google map.
